# Boron-Rich Boron Carbide Nanoparticles as a Carrier in Boron Neutron Capture Therapy: Their Influence on Tumor and Immune Phagocytic Cells

**DOI:** 10.3390/ma14113010

**Published:** 2021-06-02

**Authors:** Dawid Kozień, Bożena Szermer-Olearnik, Andrzej Rapak, Agnieszka Szczygieł, Natalia Anger-Góra, Janusz Boratyński, Elżbieta Pajtasz-Piasecka, Mirosław M. Bućko, Zbigniew Pędzich

**Affiliations:** 1 Faculty of Materials Science and Ceramics, AGH University of Science and Technology, 30 Mickiewicz Av., 30-059 Kraków, Poland; bucko@agh.edu.pl (M.M.B.); pedzich@agh.edu.pl (Z.P.); 2 Hirszfeld Institute of Immunology and Experimental Therapy, Polish Academy of Sciences, 53-114 Wroclaw, Poland; bozena.szermer-olearnik@hirszfeld.pl (B.S.-O.); andrzej.rapak@hirszfeld.pl (A.R.); agnieszka.szczygiel@hirszfeld.pl (A.S.); natalia.anger@hirszfeld.pl (N.A.-G.); janusz.boratynski@hirszfeld.pl (J.B.); elzbieta.pajtasz-piasecka@hirszfeld.pl (E.P.-P.)

**Keywords:** boron-rich boron carbide nanoparticles, functionalization, boron neutron capture therapy

## Abstract

The aim of the work was to study the interaction between boron-rich boron carbide nanoparticles and selected tumor and immune phagocytic cells. Experiments were performed to investigate the feasibility of the application of boron carbide nanoparticles as a boron carrier in boron neutron capture therapy. Boron carbide powder was prepared by the direct reaction between boron and soot using the transport of reagents through the gas phase. The powder was ground, and a population of nanoparticles with an average particle size about 80 nm was selected by centrifugation. The aqueous suspension of the nanoparticles was functionalized with human immunoglobulins or FITC-labeled human immunoglobulins and was then added to the MC38 murine colon carcinoma and to the RAW 264.7 cell line of mouse macrophages. Flow cytometry analysis was used to determine interactions between the functionalized boron carbide nanoparticles and respective cells. It was shown that B_4_C–IgG nanoconjugates may bind to phagocytic cells to be internalized by them, at least partially, whereas such nanoconjugates can only slightly interact with molecules on the cancer cells’ surface.

## 1. Introduction

Boron carbide is a covalent material characterized by a high melting point, relatively low density, and high chemical resistance; when in the form of dense polycrystals, it is characterized by a high Young modulus, extreme hardness, and good mechanical properties [1,2,3]. Such properties are suitable for boron carbide applications in components resistant to abrasion in ball mills, nozzles, parts of machinery, and equipment and as parts of antiballistic armor. Due to the high neutron absorption cross section, boron carbide is used as an absorbing and screening material in the nuclear industry [4].

Recently, boron carbide was newly applied in medical applications, for example, as a carrier in boron neutron capture therapy (BNCT) [5]. This therapy uses the neutron capture reaction of the nuclei of non-radioactive boron, ^10^B, which results in the unstable nucleus ^11^B, which decays into an α particle and a recoiled lithium nucleus, ^7^Li. The effects of this therapy strongly depend on delivery of boron compounds into tumor cells, followed by their irradiation with low energy epithermal neutrons. Currently, two metalloorganic substances have been used in clinical trials as boron carriers, sodium borocaptate and L-boronophenylalanine, but neither agent accumulates specifically in tumor tissue, and the amount of ^10^B they carry does not provide an effective therapeutic dose [6,7]. The introduction of nanoparticles to an inorganic compound carrying a high amount of boron, e.g., boron carbide, into the tumor cell might overcome this problem.

Two stoichiometries have been proposed to describe the stable phases of boron carbide. The first one is the carbon-rich B_4_C (or B_12_C_3_) compound, with a crystal structure consisting of 12-atom icosahedral boron units joined by linear three-atom carbon chains [8]. The second one is the boron-rich B_13_C_2_ (or B_6.5_C) compound, described by a (B_12_) CBC structural formula, where the central carbon atom in the carbon chain is replaced by boron [9]. In the B–C system, a wide range of compositions, from 8.8 to 20 at % C, is used to form thermodynamically stable solid solutions. Beyond ∼20 at % C, a mixture of boron carbide and graphite is often observed; below ∼8 at % C, pure boron coexists with boron carbide. Several theoretical and experimental studies have suggested that the atomic bonding, electron density, mechanical properties, and lattice constants of boron carbide solid solutions change with boron to carbon ratios. The structures of both stable boron carbide phases and solid solutions in the B–C system are described by the rhombohedral symmetry with the R3m space group. The existence of some other boron carbide phases, such as a monoclinic modification of B_13_C_2_ and tetragonal B_50_C_2_, was also reported [10].

Boron carbide powders can be synthesized via many different methods. The most popular method is the carbothermal or magnesiothermic reduction of boric acid at a temperature over 2000 °C, but the powders produced by this method require intensive grinding and purification [11]. Fine-grained boron carbide powders can be obtained by the direct reaction between B_2_O_3_ or H_3_BO_3_ and organic carbon precursors, such as saccharose, cellulose, glycerine, and poly(vinyl) alcohol [12,13]. Such methods are time-consuming and labor-intensive and require temperatures over 1600 °C, and carbon becomes an unavoidable impurity.

Boron carbide powders are rarely prepared via direct synthesis from the elements. Such synthesis is more efficient and can occur in the temperature range of 1200–1500 °C if reactive carbon forms, such as carbon nanotubes [14], amorphous carbon [15], or expanded graphite [16], are used as carbon substrates or when advanced synthesis techniques, such as CVD [17] or mechanical alloying [18], are used. Good results, especially for the synthesis of boron-rich fine nanoparticles, are obtained from the application of amorphous boron and soot in the form of loose beds where the transfer of both reagents through a gas phase occurs.

Recently, there have been reports on the use of boron carbide in BNCT therapy. One such research method consists of the treatment of oral squamous cell carcinoma with B_4_C particles encapsulated in a graphitic layer with an approximately 200 nm size, which resulted in the decrease in the survival of cells in vitro and the inhibition of tumor growth in vivo after exposure to a neutron beam [5,19]. Another in vitro study showed that murine EL4 thymoma cells and B16 F10 malignant melanoma cells loaded with coated boron carbide nanoparticles prior to exposure to neutron irradiation were able to inhibit the proliferative capacity of untreated neighboring cells [19]. In another example, B16-OVA tumor cells were exploited. These cells were preincubated with boron nanoparticles and injected subcutaneously into C57BL/6J mice and further exposed to neutron radiation. As a result, a tumor growth delay was found, compared with the untreated control mice [20]. Likewise, a Japanese group researched the internalization of submicrometric boron carbide particles into cancer cells via the surface; the transferrin conjugation revealed that the internalization of large boron-rich particles may be associated with a lower dose in achieving a therapeutic effect [21]. Another approach to the functionalization of nanoparticles (NPs) aimed at the effective delivery of boron to the affected area; the NPs were combined with antibodies [22]. Despite the growing number of publications reporting the possibilities of using the various boron-rich nanoparticles as potential carriers in BNCT, many problems remain to be overcome. One of them is the relationship between the physicochemical properties of boron carbide (size and shape) and its biological properties (the ability to bind to a target cell). For this purpose, we decided to analyze the influence of boron carbide nanoparticles, synthesized using the vapor transfer of the reagents, on the uptake by tumor and immune phagocytic cells as a potent method of direct delivery into tumor tissue. Given the growing amount of other data as well as our own results, boron-rich boron carbide nanoparticles may be suitable for application in boron neutron capture therapy.

## 2. Materials and Methods

### 2.1. Synthesis and Characterization of Boron Carbide

Boron (Fluka 15580) and soot (Tujmazy P-803) powders were used as reagents. The powders were mixed in a boron to carbon molar ratio of 4:1. The mixed powders were heat-treated at 1700 °C for 2 h under argon flow. After the synthesis, a part of the powder was separated and subjected to grinding in propanol for 24 h in a rotary vibratory mill. Finally, two populations of nanoparticles were selected by centrifugation (MPW-341, MPW Med. Instruments, Poland of water suspension of the prepared powder during 45 min and 1.5 h.

X-ray diffraction was used for the qualitative and quantitative phase analysis of the starting and final powders (Empyeran, Panalitycal, UK)). The Rietveld refinement was used to calculate the amount of the detected phases, whereas the Scherrer formula was used to estimate the crystallite size. The morphology of the nanoparticles was characterized by scanning (Nova NanoSEM 200, FEI Company and SU-70, Hitachi, Hillsboro, OR, USA) and transmission electron microscopy (JEM 1011, Jeol, Tokyo, Japan). Grain-size distribution was determined using the laser light diffraction method (Zetasizer, Malvern, UK).

### 2.2. Preparation of FITC-Labeled Antibodies Conjugated with Boron Carbide Nanoparticles

We mixed 1 mL of 0.1% boron carbide nanoparticles suspension (0.3 mg/mL) in 10 mM borate buffer (pH 8.0) with 10 μg of human immunoglobulin (Sigma–Aldrich, St. Louis, MO, USA) or FITC-labeled human immunoglobulins. The suspension was incubated at 4 °C for 12 h and then centrifuged for 10 min at 20,000× *g*. The pellet was washed twice with phosphate-buffered saline (PBS) containing 0.1% bovine serum albumin (BSA). For final experiments, nanoparticles were suspended in PBS.

### 2.3. Immunoassay of the Boron Carbide Nanoparticles on Glass Microfiber Paper

One microliter of 0.01% boron carbide nanoparticles with human immunoglobulin in PBS was spotted on Whatman 934-AH Glass Microfiber filters. After drying, the paper was blocked with 1% casein in the tris-NaCl-buffered saline (TBS) for 1 h and then incubated with 1 μg/mL protein A/G labeled with horseradish peroxidase (HRP, Thermofisher Scientific, Rockford, IL, USA) for 1 h at room temperature. After washing with TBS, the paper was incubated with a chromogenic substrate for the tetramethylbenzidine membrane (TMB, Sigma–Aldrich, St. Louis, MO, USA) and, finally, washed with water.

### 2.4. Cell Culture

The in vivo growing of MC38 murine colon carcinoma from the Tumor Bank of the TNA Radiobiology Institute, Rijswijk, Netherlands, was adapted to in vitro conditions as described by Pajtasz-Piasecka et al. [23]. The MC38/0 cell culture was maintained in RPMI 1640 (Gibco, Life Technologies Limited, Paisley, UK), supplemented with 100 U/mL penicillin, 100 mg/mL streptomycin, 1 mM sodium pyruvate, 2-mercaptoethanol, and 5% fetal bovine serum (FBS; all from Sigma–Aldrich, St. Louis, MO, USA). In the performed experiments, 1 × 10^5^ cells/mL/well was placed in a 24-well plate (Corning Costar, New York, NY, USA).

The RAW 264.7 cell line of mouse macrophages obtained from ATCC^®^ (TIB-71^TM^) were cultured in DMEM medium (ATCC, Manassas, VA, USA) supplemented with 10% FBS (Sigma–Aldrich St. Louis, MO, USA). In the performed experiments, 5 × 10^4^ cells/mL/well was placed in a 24-well plate (Corning Costar, New York, NY, USA).

The compounds containing boron carbide nanoparticles conjugated with FITC-labeled antibodies (B_4_C–IgG–FITC), and boron carbide nanoparticles without antibodies (B_4_C); antibodies, alone (IgG); and FITC-labeled antibodies (IgG–FITC) were added to MC38/0 and RAW 264.7 cell cultures at a concentration of 50 μL/well. Both tumor and macrophage-like cells were exposed to the compounds for 4 and 24 h; afterward, they were collected for flow cytometry analysis. During flow cytometry analyses, DAPI dye (Molecular Probes, Eugene, OR, USA) was used to eliminate the dead cells.

All cell cultures were performed in a NUAIRE CO_2_ incubator (95% humidity, 37 °C, 5% CO_2_). For the fluorescence intensity analysis, a FACS Fortessa flow cytometer with Diva software (Becton Dickinson, version 8.0.2, San Jose, CA, USA) was used. The cytometric data presentations were prepared using NovoExpress software (Agilent, version 1.3.0, Santa Clara, CA, USA).

## 3. Results

The X-ray diffraction analysis of the precursor powders showed that the soot powder is composed of amorphous carbon, only, whereas the boron powder is composed of rhombohedral alpha boron (R-3m, >90 mass %), a small amount of the tetragonal boron (P4_2_/nnm, ~9 mass %), and ~1 mass % of boric acid. The SEM observations showed that the soot powder is strongly aggregated: the smallest particles are less than 100 nm in size, and the bigger aggregates reach 50 μm. The boron powder is composed of crystallites with an average size ~800 nm, and aggregates are smaller than 10 μm.

The examination of the boron–carbon bed after the synthesis revealed the presence of three layers, differing from each other in color and consistency; therefore, each layer was separately subjected to X-ray diffraction analysis. The XRD analysis showed that the powder from the layer located on the top of the bed is composed of a graphite phase, formed from the amorphous soot during heat treatment. The powder from the intermediate layer is composed of a two-phase rhombohedral boron carbide with a stoichiometry close to that of B_13_C_2_ and a small amount of graphite. In the powder forming the layer located at the bottom of the crucible, three phases were detected: a rhombohedral boron carbide phase (~70 mass %), a tetragonal boron carbide phase with formal stoichiometry B_48_(B_2_C_2_) (~30 mass %), and a small amount of the graphite phase (~1 mass. %). The unit cell parameters of the rhombohedral boron carbide in this layer are a = 5.5865 A, c = 12.0876 A, and c/a = 2.1637; this suggests that the carbon content in this phase is ~14 at %, which corresponds to stoichiometry close to that of B_6_C. The presence of boron carbides phases in each layer indicates the possibility of transferring reagents through the gas phase: boron in the form of vapors and/or carbon in the form of carbon monoxide. This will be discussed in a separate paper.

**Figure 1:**
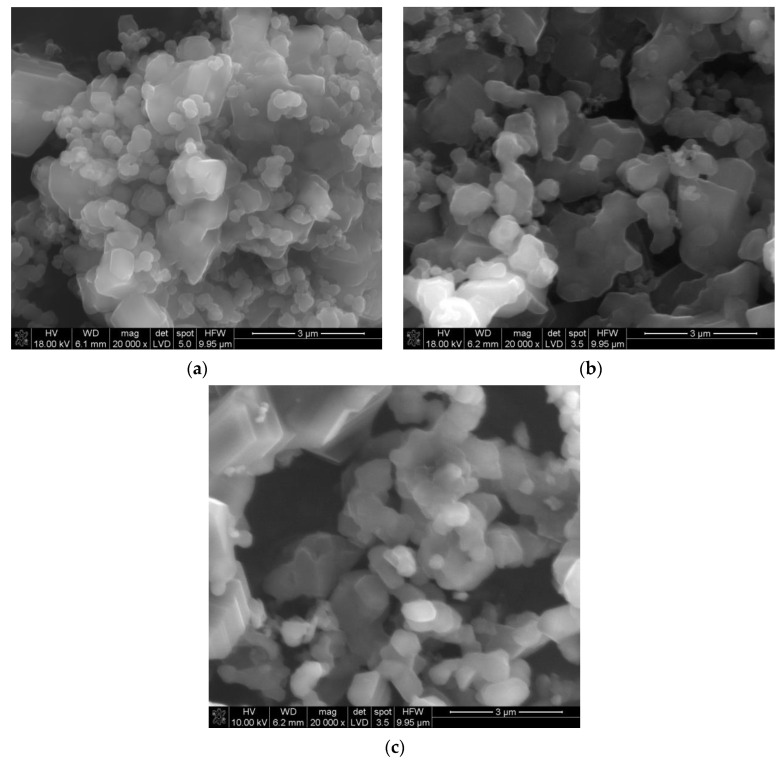
SEM image of the nonground boron carbide powder (**a**–**c**).

**Figure 2:**
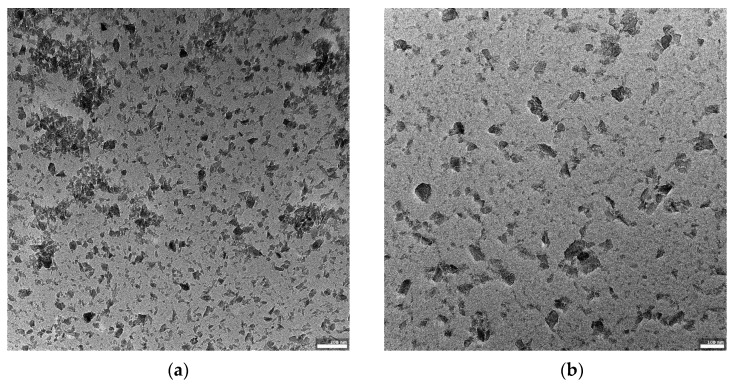

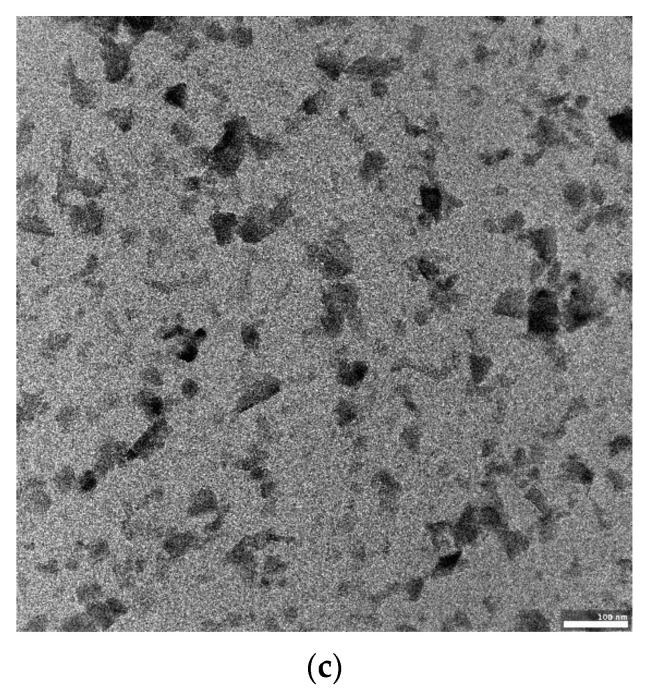
The structure and morphology of B4C powder centrifuged for 1.5 h. (**a**–**c**) present the differentiation in morphology of the obtained boron carbide nanoparticles in increasing magnification. TEM visualization using a JEOL JEM-F200 80 kV TEM (scalebars 100 and 200 nm).

Figure 1a–c shows an SEM image of the non-ground powder after synthesis. The powder is composed of crystallites, whose sizes start from a few hundreds of nanometers, joined into larger forms. The shapes of some of the smallest particles show the rhombohedral symmetry of the alpha boron phase. The grinding process caused an effective disaggregation of the powder. Additionally, Figure 2a–c shows the morphology of the obtained boron powder centrifuged for 1.5 h and visualized using transmission electron microscopy.

**Figure 3:**
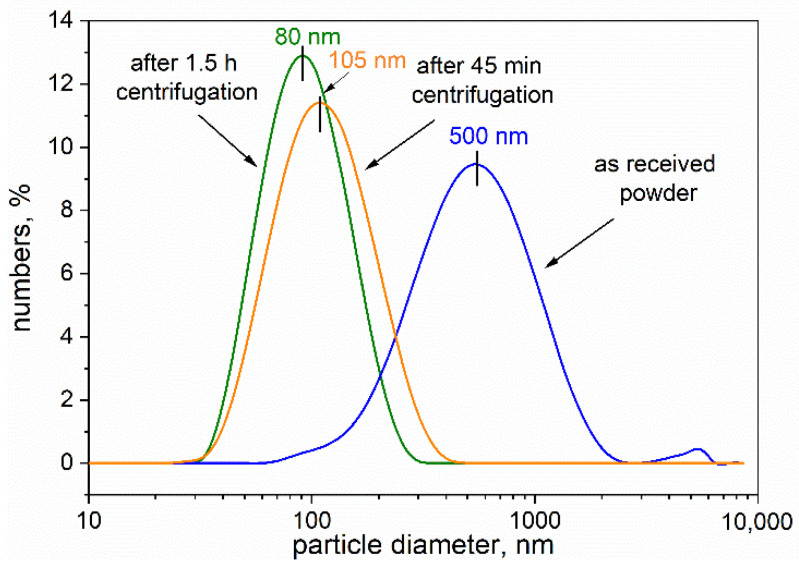
Particle size distributions of the ground boron carbide powders and the fraction after 45 min and 1.5 h centrifugation measured using dynamic light scattering technique.

Figure 3 reveals the grain size distributions of the starting ground powder and the centrifuged powders. The distributions proved to be unimodal, and the average size of the particles in the starting powder was ~500 nm, and 105 and 80 nm for particles in the powders centrifuged for 45 min and 1.5 h, respectively. These obtained data are in good agreement with the results observed by TEM. Figure 2a–c shows the population of boron carbide nanometric particles.

For biological testing, we selected a fraction after 1.5 h centrifugation because of the favorable PdI (0.020) value, which suggests higher homogeneity of nanoparticles compared with the other fractions. According to the DLS measurements, the average particle size was about 80 nm. The first stage of biological investigation involved comparing the interaction of the nanoparticles with the cells when the compounds were used in their native form or after biological functionalization. For this purpose, the RAW264.7 phagocytic cells were exposed to native boron-rich compounds or the same compound previously coated with IgG antibodies.

**Figure 4:**
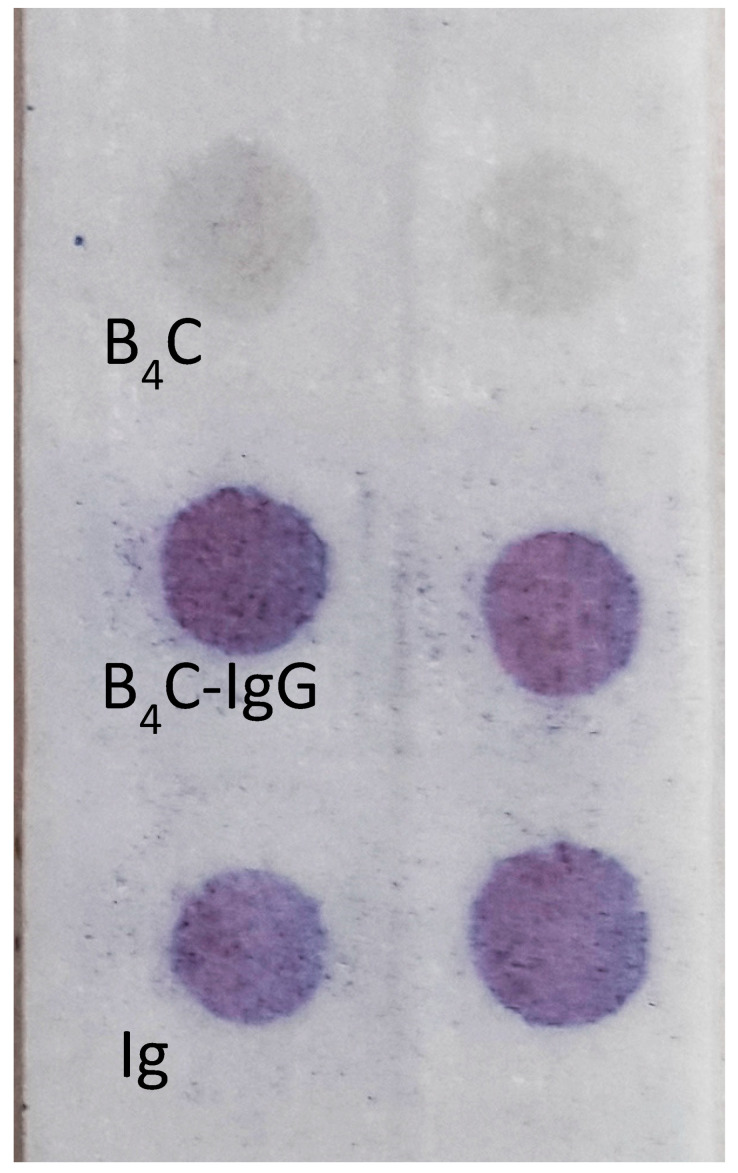
Immunoenzymatic test on 934-AH glass microfiber paper. The presence of immunoglobulin G was detected with protein A/G–peroxidase conjugate. The negative reaction of boron carbide nanoparticles (B_4_C), alone, and the positive result of boron carbide nanoparticles conjugated with human immunoglobulins (B_4_C–IgG), and the positive control human immunoglobulins, alone (IgG). The experiments were performed in duplicate.

To confirm whether the boron carbide nanoparticles were able to adsorb stably on the surface of human immunoglobulin G (IgG), we set up a spot test on glass microfiber paper. A small drop of B_4_C–IgG suspension was spotted on glass paper, then the presence of the adsorbed immunoglobulin was detected enzymatically with protein A/G labeled with peroxidase. The obtained results revealed the presence of human IgG on the surface of the boron carbide nanoparticles (Figure 4) and confirmed the possibility of using this conjugate for the subsequent analysis of nano-B_4_C uptake by phagocytic cells. The effectiveness of the B_4_C–IgG was tested using a dot immunoassay for 12 months, which showed that the complex is stable.

**Figure 5:**
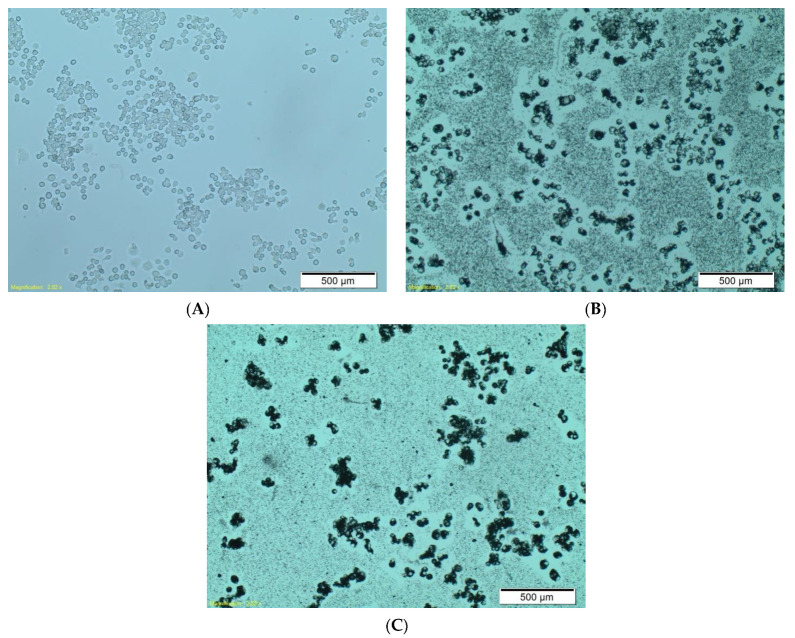
The interaction of RAW264.7 phagocytic cells with boron carbide nanoparticles functionalized by coating with human IgG (**C**). Untreated control cells (**A**) and boron carbide nanoparticles without coating IgG (**B**) after 24 h incubation. We used an Olympus CKX41 light microscope, magnification 6.3×.

As mentioned above, we examined the ability of RAW264.7 phagocytic cells to uptake the functionalized boron carbide nanoparticles. The effect was clearly visible under a light microscope, where we compared the interaction of boron carbide nanoparticles, alone (Figure 5b), with that of nanoparticles coated with human IgG (Figure 5c). This observation revealed a much stronger interaction of the human IgG–B_4_C nanoconjugate with RAW264.7 cells than with native nanoparticles. We postulate that the phenomenon observed in Figure 5c may be related to the electrostatic interaction between the nanoparticles and the cell membrane.

**Figure 6:**
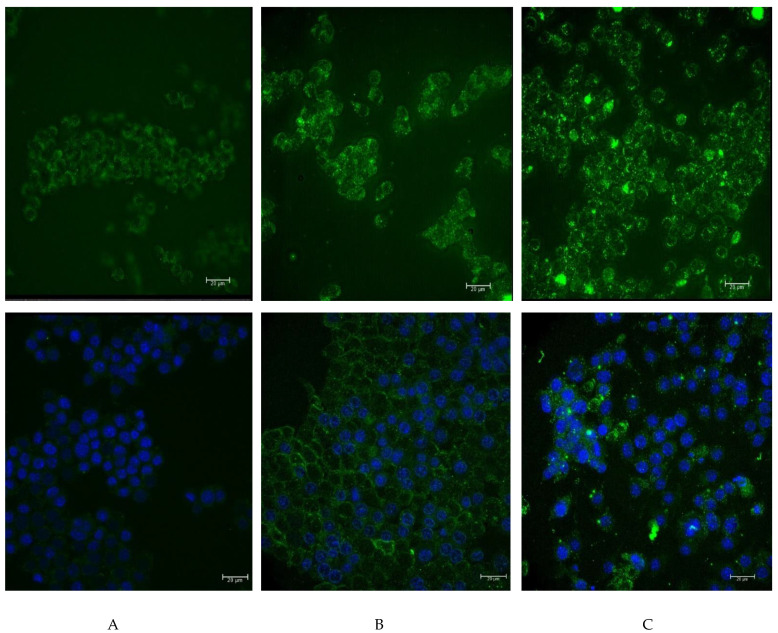
The interaction of RAW264.7 phagocytic cells with the boron carbide nanoparticles functionalized by coating with human IgG labeled with FITC. The microscopic image was obtained using the Leica THUNDER Imaging System. (**A**) The control RAW264.7 cells; (**B**) the RAW264.7 cells after 24 h incubation with FITC-labeled human IgG; (**C**) the RAW264.7 cells after 24 h incubation with the boron carbide particles coated with the FITC-labeled human IgG. The top line shows the control cells and cells after incubation with boron nanoparticles containing FITC-labeled antibodies. The lower line shows the paraformaldehyde-fixed cell preparations with the nuclei additionally stained using DAPI (scalebars 20 µm).

To analyze the possibility of functionalized nanoparticles being internalized, we decided to label them with fluorochrome. For this purpose, the nanoparticles were coated with FITC-labeled human IgG and were then added to RAW264.7 cells. The background noise resulting from the autofluorescence of cells is presented in Figure 6a. Figure 6b presents the weak glow of the FITC-labeled human IgG. From the analysis of the effects of 24 h incubation, we found a noticeable interaction of the boron-rich carrier with phagocytic cells (Figure 6c). The results suggested that the nanoparticles were attached to the surface of RAW264.7 cells, and that they can be internalized by these cells. Additional staining with DAPI agrees with the results obtained on the unfixed material (Figure 6, lower line).

**Figure 7:**
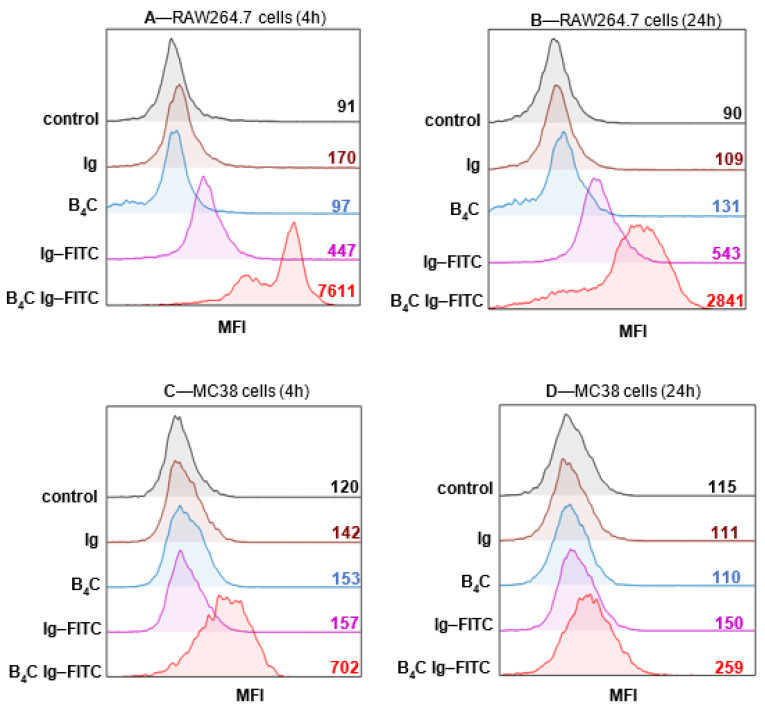
Evaluation of the interaction level of boron carbide nanoparticles with myeloid and cancer cells. Mean fluorescence intensity (MFI) was analyzed using flow cytometry after 4 and 24 h of exposure of RAW264.7 (**A**,**B**) and MC38/0 cells (**C**,**D**) on the labeled carrier. IgG, human immunoglobulin; IgG–FITC, fluorescein-labeled IgG; B_4_C, boron carbide nanoparticles; B_4_C Ig–FITC, boron carbide nanoparticles functionalized with human FITC-labeled IgG.

We concluded that the level of cellular uptake of boron-rich nanoparticles should be further evaluated using a specific assay. As such, we exploited flow cytometry for the analysis of the interaction of boron carbide with the target cells. The effectiveness of protein binding was determined using the mean fluorescence intensity (MFI), which is presented in representative histograms. The obtained findings confirmed that the immune cells, RAW264.7 phagocytic cells, were able to recognize and bind boron carbide particles conjugated with IgG–FITC (B_4_C IgG–FITC) (Figure 7A,B). An additional experiment was conducted on the cells of the MC38/0 colon carcinoma line, chosen to define whether the cancer cells would be able to interact with the boron-rich nanoparticles. These cells were able to bind the tested particles (Figure 7C,D), and the response of the tumor cells was much lower than that of the phagocytic cells. As a result of the investigation, the differences in the level of boron uptake were found to depend on the length of exposure time, and an incubation period time shorter than 4 h resulted in higher MFI in both types of cells, compared with the 24 h exposure time. This observation indicated that uptake the boron-rich nanoconjugates occurs most efficiently in a short time, proving that the recognition and internalization of functionalized nanoparticles are quick processes and are sufficient to deliver these particles to target cells.

## 4. Discussion

Two boron-containing drugs are currently applied clinically: boronophenylalanine (BPA) and sodium borocaptate (BSH). Their therapeutic efficacy has been demonstrated in patients with high-grade malignancies. Due to therapeutic limitations of these drugs, many efforts have been devoted to the development of new boron delivery agents with more beneficial biodistribution and cellular uptake. These include low-weight, organic, boron-rich compounds, monoclonal antibodies or liposomes, as well as tubes or spherical native boron compounds belonging to a new form on nanoparticles [7].

We proposed boron-rich boron carbide nanoparticles in this paper, obtained by direct synthesis using the transport of reagents through the gas phase; we additionally employed unique methods of biofunctionalization to fabricate a new form of boron carriers. To confirm their wide capability range, we performed in vitro tests to verify whether the synthesized boron carbide nanoparticles can effectively interact with phagocytic and tumor cells.

The growing amount of evidence is showing that tumors are a complex biological systems consisting of cancer cells and various auxiliary cells, within stromal cells, directly linked to the remodeling and promotion of tumor growth, together creating a tumor microenvironment [24]. Due to the abundance of tumor-associated macrophages (TAMs) in the cancerous environment, these cells are an interesting target for nanoparticles of different sizes. Macrophages efficiently engulf both large and small nanoparticles using a variety of different receptors of immunoglobulins and lectins or scavenger receptors, which all belong to the system of early recognition of different modified and functionalized molecules. In contrast with phagocytic cells, most epithelium-derived tumor cells use pinocytosis instead of phagocytosis. Therefore, in this study, we examined the influence of boron carbide nanoparticles in their native form and after functionalization by human IgG on the binding and/or uptake efficiency of two types of cells: RAW264.7 phagocytic cells, considered carriers of boron-rich compounds, and MC38/0 cells, as a direct target for boron nanoparticles in BNCT.

An important step in cellular uptake is the physical interaction between the nanoparticles and the cell membrane. This interaction can lead to the segregation and clustering of nanoparticles on the cell surface [25]. It was shown that nanoparticles can disturb the functionality and integrity of cellular membranes in a size-dependent manner [26]; therefore, at the beginning of study, we repeatedly obtained several fractions with a defined particle size of about 100 nm. The particle size distribution and the TEM images revealed that the fraction of smaller size particles was prepared after centrifuging for 1.5 h. Such parameters classified those particles for the evaluation of cellular uptake. No cytotoxic effect was found for those particles with the concentration used in the experiment on either phagocytic or tumor cells (data not shown).

Subsequently, we attempted to functionalize these nanoparticles with human immunoglobulin. Similar investigations were previously performed with proteins and other organic molecules [27,28]. Our findings confirmed that the nanoparticles we prepared are able to associate with proteins, represented by human IgG. When we compared the stage of binding of the native and functionalized agents (nanoconjugate B_4_C with IgG, B_4_C IgG–FITC), we observed a visible increase in uptake of the latter. The strong interaction of B_4_C IgG–FITC manifested in the restriction on phagocytic cells (MC38/0 cells), which were also able to interact with this nanoconjugate, yet their response was much lower than that of phagocytic cells. Moreover, a higher fluorescence signal was detected after a shorter incubation time, most likely due to degradation of labeled IgG by the proteasomal and/or lysosomal system.

The critical parameters controlling nanoparticles’ uptake by phagocytic cells are dependent on particle and cellular surface properties [29]. Both nanoparticles with charged and hydrophobic surfaces have a tendency to be attractive for complementary proteins or other opsonins; as such, they are more effectively captured by phagocytic cells [30]. The known dominant role of electrostatic over hydrophobic interactions between nanoparticles and the lipid bilayers of cellular membranes may be crucial when considering cells covered by native boron carbide compounds [31]. Moreover, the size and shape of tested nanoparticles were found to influence the level of uptake by different cells, according to data that particles smaller than 50 nm are much easier to internalize than larger particles [32]. However, in the case of larger nanoparticles, their functionalization leads to increased, stability-facilitating, effective uptake. Since most antibodies have a high affinity for hydrophobic particle surfaces, we harnessed this feature to bind human immunoglobulin on the surface of boron carbide. In our study, IgG–B4C conjugate was planned for cell loading in in vitro cultures for their future use as a carrier directly applied to a tumor-bearing host.

## 5. Conclusions

We postulated that B_4_C–IgG nanoconjugates may selectively and directly bind to RAW 246.7 cells to be internalized by them. We examined the usefulness of phagocytic cells for accumulating boron-rich nanoparticles as potent carriers in BNCT. It seems to be especially important in the case where tumor cells, such as those tested in our study (MC38 cells), are able to only slightly interact with analyzed compound. Therefore, further studies on the level of nanoconjugate uptake need to be performed.

## Data Availability

The data presented in this study are available on request from the corresponding author.
